# Toll-like receptors expression and interferon-γ production by NK cells in human sepsis

**DOI:** 10.1186/cc11838

**Published:** 2012-10-25

**Authors:** Fernando Souza-Fonseca-Guimaraes, Marianna Parlato, François Philippart, Benoît Misset, Jean-Marc Cavaillon, Minou Adib-Conquy

**Affiliations:** 1 Unité Cytokines & Inflammation, Département Infection et Epidemiology, Institut Pasteur, 28 rue Dr. Roux, 75015 Paris, France; 2 Groupe hospitalier Paris Saint Joseph, Medical and surgical Intensive care unit, Paris Descartes University, Paris, France

## Abstract

**Introduction:**

During the course of infection, natural killer (NK) cells contribute to innate immunity by producing cytokines, particularly interferon-gamma (IFN-γ). In addition to their beneficial effects against infection, NK cells may play a detrimental role during systemic inflammation, causing lethality during sepsis. Little is known on the immune status of NK cells in patients with systemic inflammatory response syndrome (SIRS) or sepsis in terms of cell surface markers expression and IFN-γ production.

**Methods:**

We investigated 27 sepsis patients and 11 patients with non-infectious SIRS. CD56^bright ^and CD56^dim ^NK cell subsets were identified by flow cytometry and Toll-like receptor (TLR)2, TLR4, TLR9, CX3CR1, CD16 and CD69 expression were analyzed, as well as *ex vivo *IFN-γ production by NK cells in whole blood samples.

**Results:**

We first showed that in NK cells from healthy controls, TLR2 and TLR4 expression is mainly intracellular, similarly to TLR9. Intracellular levels of TLR2 and TLR4, in both CD56^bright ^and CD56^dim ^NK cell subsets from sepsis patients, were increased compared to healthy subjects. In addition, the percentage of CD69^+ ^cells was higher among NK cells of sepsis patients. No difference was observed for TLR9, CX3CR1, and CD16 expression. The *ex vivo *stimulation by TLR4 or TLR9 agonists, or whole bacteria in synergy with accessory cytokines (IL-15+IL-18), resulted in significant production of IFN-γ by NK cells of healthy controls. In contrast, for SIRS and sepsis patients this response was dramatically reduced.

**Conclusions:**

This study reports for the first time an intracellular expression of TLR2 and TLR4 in human NK cells. Surface TLR4 expression allows discriminating sepsis and SIRS. Furthermore, during these pathologies, NK cells undergo an alteration of their immune status characterized by a profound reduction of their capacity to release IFN-γ.

## Introduction

Both severe sepsis and systemic inflammatory response syndrome (SIRS) are characterized by an exacerbated inflammatory response and a cytokine storm [1]. Concomitantly, sepsis and SIRS patients undergo a compensatory anti-inflammatory response syndrome (CARS) [2]. This modification of immune status, also called reprogramming, has been regularly reported for circulating monocytes, neutrophils and lymphocytes in sepsis [3-5]. This alteration is also characterized by reduced HLA-DR expression on monocytes [6]. Leukocytes from septic patients respond poorly to Toll-like receptor (TLR) agonists compared to cells from healthy subjects [7-9]. In many aspects, this hypo-reactivity resembles the well-known phenomenon of endotoxin tolerance, which is characterized by lack of response to a second challenge with lipopolysaccharide (LPS) *in vivo *or *in vitro*, given shortly after a first exposure to LPS [7]. The concept has been extended to all TLR agonists.

Although the immune status of monocytes, lymphocytes and neutrophils is well-characterized in SIRS and sepsis patients, little is known about that of natural killer (NK) cells in these patients. In humans, at least 2 subsets of circulating NK cells have been described, the CD3^-^CD56^dim ^and CD3^-^CD56^bright^. These two subsets do or do not express other cell surface markers (for example, CCR7, CD25, CD117), and show differential function (for example, CD56^dim ^NK cells display enhanced cytotoxicity; CD56^bright ^NK cells produce greater amounts of cytokines) [10]. NK cells are a major source of IFN-γ, a cytokine known to activate monocytes and macrophages, and to contribute to immune responses against bacterial infections [11,12]. However, experiments performed with recombinant IFN-γ, neutralizing antibodies, or IFN-γ-receptor-deficient mice, established that this cytokine is also a key contributor to lethality after LPS injection or in sepsis models [13-15]. Circulating IFN-γ is found after LPS injection in mice [16] and in murine models of polymicrobial sepsis [17], as well as in patients with sepsis [18]. *In vivo *induction of endotoxin tolerance is associated with a dramatic reduction of circulating IFN-γ [16]. IFN-γ is also known to prevent endotoxin tolerance of monocytes and macrophages [19], and has been shown to reverse the altered immune status of monocytes in human sepsis [20]. Similarly, beneficial and deleterious roles of NK cells have been reported during bacterial infection, (for review see [21]). Protective functions of NK cells have been reported in septic peritonitis and in lung infection with Gram-positive bacteria. In contrast, deleterious roles of NK cells have been reported in animal models after polymicrobial intra-abdominal sepsis, *Escherichia coli *intraperitoneal injection, *Streptococcus pyogenes *intravenous injection, cytokine-induced SIRS, and in a polytrauma model.

Little is known about the specific responsiveness of human NK cells to pathogen-associated molecular patterns (PAMPs), although they do express mRNA encoding for TLRs [22-24]. It has been reported that after LPS or bacterial DNA challenge, most IFN-γ-producing cells were NK cells [25,26]. Endotoxin (a TLR4 agonist), flagellin (a TLR5 agonist), and outer membrane protein A (a TLR2 agonist) alone are each weak stimuli for NK cells of healthy volunteers, whereas in the presence of accessory cytokines (for example, IL-2, IL-15, IL-12, or IL-18) large amounts of IFN-γ are produced [23,27]. Similarly, NK cells are able to respond to nucleotide oligomerization domain receptor 2 (NOD2) agonists [28] or endogenous danger signals such as high-mobility group, box 1 [29]. However, the capacity of NK cells to respond to TLR agonists has not yet been characterized in SIRS and sepsis patients. In sepsis patients, a decrease in the number of circulating NK cells has been regularly reported [30,31]. When NK cells were functionally studied, their cytotoxic activity was found to be reduced in sepsis patients [32,33] and also in SIRS patients following thermal and traumatic injury [34,35]. Very few studies have addressed the capacity of NK cells in sepsis or SIRS patients to produce IFN-γ. Additionally no study has shown the expression levels of the early activation marker CD69 [36] on the surface of this cell type in these patients. It has been shown that the production of IFN-γ in response to IL-2 and IL-12 in combination (with or without IL-18) is altered in patients who undergo elective surgery and severely impaired in patients with sepsis [37]. However, to date, no study has addressed the responsiveness of NK cells to TLR agonists in human SIRS and sepsis.

This report investigates the modulation of cell surface markers (activation markers and receptors), and for the first time, TLR expression on NK cells of SIRS and sepsis patients. Furthermore, we investigated the capacity of NK cells to produce IFN-γ in the presence of TLR agonists and stimulatory cytokines, to investigate whether these cells could exhibit endotoxin tolerance as previously shown for monocytes. In addition, we extended our study by analyzing the two NK cell subsets (CD56^bright ^and CD56^dim^).

## Materials and methods

### Patients

Patients included in this study belong to the combined approach for the early diagnosis of sepsis (CAPTAIN) cohort (ClinicalTrial, n°NCT01378169) and were from all seven ICUs involved in this protocol. The CAPTAIN study is an ongoing observational multicenter investigation aimed to define the best combination of biomarkers (plasmatic microbial or host markers; leukocyte surface markers) for an early diagnostic of sepsis in ICU patients. All patients receive conventional therapy and care. The study was approved by the regional ethical committee on 19 November 2010 (*Comité de Protection des Personnes - Ile de France XI*, number: 2010-A00908-31). Written informed consent to participate in the study was obtained for each patient, or if impossible, from the patient's next-of-kin. Presence of SIRS and suspicion of infection were the only criteria for inclusion. Intensive care patients with SIRS were included within 24 hours after medical staff suspected infection. Practically, this was when patients had fever associated with biological parameters (increase in white blood cells, CRP level) and a suspected infection site. Infection could be community- or hospital-acquired and the blood sample could be taken just after ICU admission or at any time during the ICU stay. Immunocompromised individuals were excluded (defined as individuals with ongoing cancer, hematologic malignancy, bone marrow or peripheral stem cell transplants, or AIDS), as were individuals undergoing chemotherapy, immunosuppressive therapy, or using steroids (> 10 mg a day for more than 15 days). Thirty-eight patients were included in this ancillary study. Individuals in whom infection was confirmed were classified as sepsis patients. Individuals in whom infection was not confirmed were classified as SIRS patients with SIRS of non-infectious origin. Patients were compared to ten healthy volunteers (Etablissement Français du Sang, EFS/Groupe Hospitalier Pitié-Salpetrière).

### Reagents

Labeled antibodies against the following antigens were used: (VioBlue)-anti-CD3 clone BW264/56, (APC)-anti-CD16 clone VEP13, (PE)-anti-CD56 clone AF12-7H3 from Miltenyi Biotec (Bergisch-Gladbach, Germany); (FITC)-anti-CD69 clone FN50 from BD Biosciences (San Diego, CA, USA); (A647)-anti-CX3CR1 clone 2A9-1, (A647)-anti-TLR2 clone TL2.1 from Biolegend (San Diego, CA, USA); (A647)-anti-TLR4 clone 76B357.1 and (A488)-anti-TLR9 clone 26C593.2 from Imgenex (San Diego, CA, USA). The IFN-γ secretion assay, and the Inside Stain Kit were purchased from Miltenyi Biotec. The human recombinant cytokines IL-15 and IL-18 were obtained from Miltenyi Biotec and MBL (Boston, MA, USA) respectively. The PAMPs used were lipopolysaccharide (LPS) TLRgrade from *E. coli *serotype 0111:B4 (Alexis, San Diego, CA, USA), hCpG-DNA 5'-ATAATCGACGTTCAAGAAAG-3' (Sigma-Gensys, The Woodlands, TX, USA) and heat-killed *S. aureus *(HKSA) (Calbiochem, La Jolla, CA, USA).

### *Ex vivo *culture conditions and IFN-γ assays

Because NK cells represent a small percentage of circulating leukocytes, and the volume of blood samples was limited, the experiments were performed in whole blood, and IFN-γ production by the two CD56^bright ^and CD56^dim ^NK cell subsets was analyzed by flow cytometry. For the IFN-γ secretion and CD69 expression assays, blood samples (125 μL) were diluted 1:1 with RPMI 1640 (LONZA, Rockland, ME, USA) alone (control); or with RPMI 1640 containing IL-15 and IL-18 (IL-15/IL-18) at 10 ng/mL final each; or IL-15/IL-18 plus LPS at 100 ng/mL; or IL-15/IL-18 plus CpG-DNA at 1 μM; or IL-15/IL-18 plus HKSA at 10 μg/mL. Cultures were primed by incubating overnight (16 h) at 37°C in a 5% CO_2 _humidified incubator.

For the CD69 measurements after *in vitro *cultures, samples were washed with 5 mL of staining buffer (PBS, EDTA 2 mM and FCS 0.5%), then stained for CD3, CD56, CD69 and CX3CR1 as described below. For the IFN-γ secretion assay (Miltenyi Biotec), after overnight incubation, blood samples were washed with 5 mL of cold staining buffer then centrifuged at 300 × *g *for 5 minutes at 4°C. Blood samples were then incubated on ice for 5 minutes with 5 μL of the IFN-γ catch reagent and 40 μL of ice-cold RPMI 1640 containing 10% human serum (BioWhittaker, Walkersville, MD, USA). Then, 5 μL of IFN-γ catch reagent and 2.5 mL of warm (37°C) RPMI 1640 containing 10% human serum were added to the blood samples; the mixture was agitated at 90 rpm for 2 hours at 37°C (agitation is crucial to avoid IFN-γ capture by non-secreting cells). Finally, samples were washed with 5 mL of staining buffer and incubated with (VioBlue)-anti-CD3 and (PE)-anti-CD56 mAbs to determine NK cell subsets as described in the following section.

### Flow cytometry

NK cell surface antigens were labeled by diluting antibodies in staining buffer at the concentrations suggested by the manufacturers. All whole blood samples (100 μL) were immediately processed for multiple staining with 5 μL (VioBlue)-anti-CD3 and 5 μL (PE)-anti-CD56 antibodies to discriminate NK cell subsets (CD3^-^/CD56^bright ^and CD3^-^/CD56^dim^). The following antibodies were combined to evaluate the expression of other markers: 5 μL (APC)-anti-CD16, 5 μL (FITC)-anti-CD69, 2 μL (A647)-anti-CX3CR1, 2 μL (A647)-anti-TLR2, 2 μL (A647)-anti-TLR4, or 1.5 μL (A488)-anti-TLR9. After 20 minutes incubation in the dark at 4°C, 2 mL of lysis buffer (BD Pharm lyse) was added for at least 10 minutes at 25°C. The incubation was followed by centrifugation (300 × *g *for 5 minutes), the cells were washed with 2 mL of staining buffer, centrifuged again and then re-suspended in 500 μL of staining buffer for surface expression analysis or in fixation buffer for intracellular staining. Intracellular stain for TLR2, TLR4 and TLR9 was performed after red blood cell lysis, washing, fixation and permeabilization of the cells using Inside Stain Kit (Miltenyi Biotec), according to the manufacturer's instructions. All flow cytometry data were acquired on a MACSQuant flow cytometer (Miltenyi Biotec) and analyzed using the MACSQuantify software. Anti-TLR antibodies were previously tested on monocytes, used as positive controls (data not shown). For each antibody, an isotype control with the same fluorophore from the same manufacturer was used as negative staining control. The number of NK cells was determined by gating on CD3^-^/CD56^bright ^and CD3^-^/CD56^dim ^subsets using the MACSQuant cytometer, allowing absolute counting.

### Statistical analysis

One-way analysis of variance (ANOVA) and the Tukey post hoc test were performed for statistical analysis of flow cytometry data. A *P*-value < 0.05 was considered significant.

## Results

### Patients' characteristics

Eleven patients were classified as non-infectious SIRS, and 27 were defined as sepsis patients [38]. Patient characteristics are described in Table 1. Among sepsis patients, the lung was the primary site of sepsis in almost two-thirds of the sepsis patients. Twelve patients had Gram-positive infections. Eight patients had an isolated Gram-positive infection, three involved a Gram-positive and a Gram-negative bacterium and one a Gram-positive bacterium and Mycobacterium tuberculosis. Sixteen patients had Gram-negative infections, with one (eleven patients) or two (two patients) different bacteria or with concomittant Gram-positive infection (three patients). A total of 19 Gram-negative bacteria were identified, including *Escherichia coli *in seven patients, *Klebsiella pneumoniae *in four patients, *Hemophilus influenzae *in two patients, and *Pseudomonas aeruginosa *in two patients. In terms of Gram-positive bacteria, *Staphylococcus aureus *was found in five patients, *Streptococcus *spp. in four and *Streptococcus pneumoniae *in one patient. *Mycobacterium tuberculosis *was found in only one patient as was *Candida albicans*. More than one pathogen was found in six patients.

**Table 1:** Parameters of the studied patients

Parameter	SIRS (*n *= 11)	Sepsis (*n *= 27)
Age, y, mean (SD)	59.7 (21.0)	70.6 (14.9)

Gender, n (%)		
Male	10 (90.9%)	14 (51.8%)
Female	1 (9.1%)	13 (48.2%)
Body mass index, Kg/m^2^, mean (SD)	26.2 (6.2)	26.9 (7.2)
Comorbidity, n (%)		
Heart failure	0 (0%)	1 (3.7%)
Ischemic cardiopathy	1 (9.1%)	7 (25.9%)
COPD	3 (27.3%)	4 (14.8%)
Chronic renal failure	1 (9.1%)	3 (11.1%)
Liver failure	0 (0%)	1 (3.7%)
Diabetes	1 (9.1%)	6 (22.2%)
Cancer	2 (18.2%)	5 (18.5%)
Infection site		
Lung	NA	17 (62.9%)
Abdominal	NA	7 (25.9%)
Urinary tract	NA	2 (7.4%)
Positive blood culture	NA	7 (25.9%) (among these, one had primitive bacteremia)
Temperature, °C, mean (SD)	38.2 (2.0)	37.8 (2.1)
Heart rate, bpm, mean (SD)	127 (23)	121.9 (18.7)
Mean blood pressure, mmHg, mean (SD)	72.9 (19.7)	65.2 (23)
Respiratory rate, c/min, mean (SD)	29.8 (14.4)	30.1 (8.2)
Leukocytes, 10E9/L, mean (SD)	12.9 (4.4)	15.08 (8.4)
SOFA, mean (SD)	8.3 (3.4)	9.1 (4.1)
SAPS II, mean (SD)	41.4 (17.5)	48.3 (21.3)
McCABE classification (underlying diseases)		
Category 1, number (%)	10 (90.9%)	22 (81.5%)
Category 2, number (%)	1 (9.1%)	4 (14.8%)
Category 3, number (%)	0 (0%)	1(3.7%)
Length of stay in hospital, days, mean (SD)	30.0 (35.2)	40.2 (42.0)
Length of stay in ICU, days, mean (SD)	11.4 (10.6)	24.1 (33.6)
In-hospital mortality, number (%)	3 (27.3%)	12 (44.4%)
Microbes, number patients Gram-positive bacteria	NA	12 (among these two had Gram-positive anaerobes)
Gram-negative bacteria	NA	19
*Mycobacterium *spp.	NA	1
Fungus	NA	1
Undetermined microbial infection		1 (polymicrobial flora)

COPD, chronic obstructive airways disease; NA, not applicable; SOFA, sequential organ failure assessment; SAPS, simlified acute physiology score; SIRS, systemic inflammatory response syndrome.

### Decrease in the number of CD56^bright ^and CD56^dim ^NK cells subsets after SIRS

**Figure 1:**
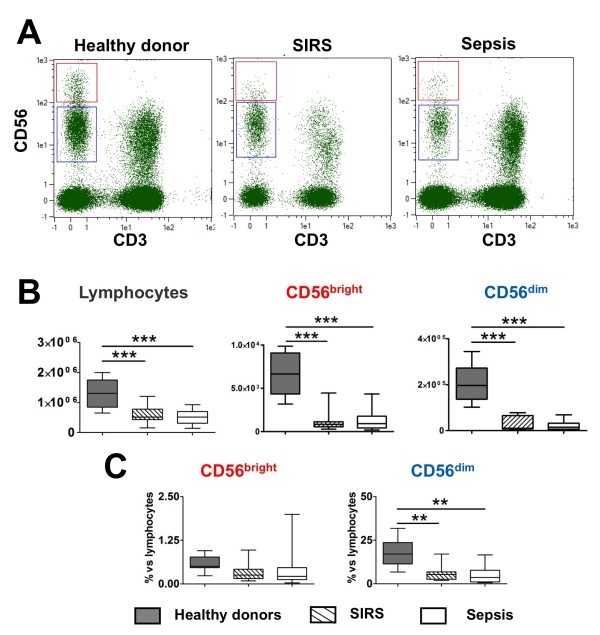
**Analysis of NK cells CD56^bright ^and CD56^dim ^subsets in sepsis and systemic inflammatory response syndrome (SIRS) patients**. (**A**) Representative chart of analysis of natural killer (NK) cells in whole blood by flow cytometry. (**B**) Cell counts for total lymphocytes and NK cell subsets (CD56^bright ^and CD56^dim^) in healthy volunteers, SIRS, and sepsis patients. (**C**) Percent of NK subsets among lymphocytes in healthy volunteers, SIRS, and sepsis patients. Data are shown as median and interquartile range. ** *P < 0.01*; ***P < 0.001.

The absolute number of NK cells was analyzed in SIRS and in sepsis patients within the first 24 hours of suspicion of infection. The number of NK cells in each subset (CD56^bright ^and CD56^dim) ^was compared between sepsis patients, SIRS patients, and healthy donors. A representative flow cytometry result for each group is shown in Figure 1A and the median and interquartile range of each group are shown in Figure 1B. It is clear that the number of NK cells in both subsets was significantly decreased in the blood of sepsis and SIRS patients, in comparison to healthy controls. No difference was found between patients with SIRS of infectious versus non-infectious etiology. These results parallel the global lymphopenia observed in ICU patients. When the number of NK cells was proportionally expressed as percent of the total lymphocyte population, the percent of CD56^dim ^NK subset was significantly decreased in both SIRS and sepsis patients (Figure 1C).

### Sepsis and SIRS modify the expression of TLR2 and TLR4 in NK cells

**Figure 2:**
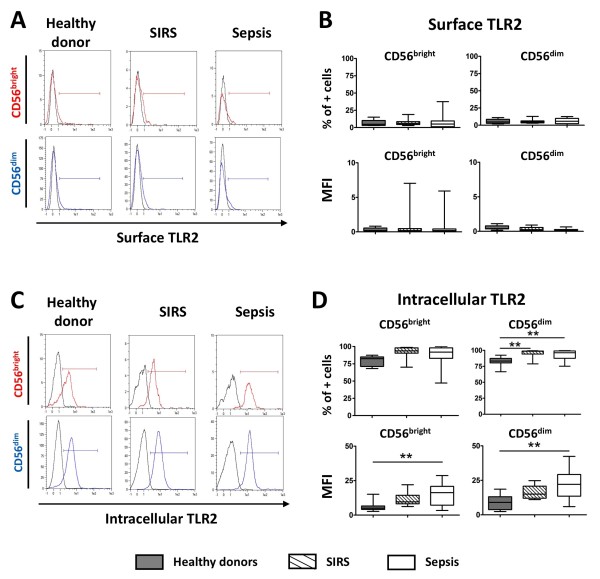
**Analysis of the expression of Toll-like receptor 2 (TLR2) by flow cytometry in natural killer (NK) cell CD56^bright ^and CD56^dim ^subsets**. (**A**) Representative flow cytometry histogram of surface expression of TLR2 on CD56^bright ^and CD56^dim ^NK cell subsets for healthy volunteers, systemic inflammatory response syndrome (SIRS), and sepsis patients. Black line: isotype control; colored line: anti-TLR2. (**B**) Surface TLR2 expression shown as median and interquartile range in all three studied groups. (**C**) Representative flow cytometry histogram of intracellular expression of TLR2 for the same samples represented in (**A)**. Black line: isotype control; colored line: anti-TLR2. (**D**) Intracellular TLR2 expression shown as median and interquartile range in all three studied groups. **P < 0.01 for comparison with healthy controls. MFI, mean fluorescence intensity.

We then analyzed the expression of TLR2 and TLR4 on and within NK cell subsets. Most of our knowledge about TLR expression in NK cells relies on mRNA analysis. Furthermore, both mRNA [22-24] and protein surface expression on human NK cells [39,40] remain controversial. We found that surface expression of TLR2 was barely detectable on both CD56^bright ^and CD56^dim ^subsets of NK cells for all groups both in terms of percentage of positive cells and mean fluorescence intensity (MFI) (Figure 2A-B). In contrast, a strong intracellular expression of TLR2 was detected after cell permeabilization in both subsets in healthy donors, as well as in SIRS and sepsis patients (Figure 2C-D). The percentage of intracellular TLR2-positive CD56^dim ^NK cells was significantly increased in sepsis and SIRS patients compared to healthy controls. Interestingly, the MFI for TLR2 in both CD56^bright ^and CD56^dim ^was significantly increased for sepsis patients compared to healthy volunteers.

**Figure 3:**
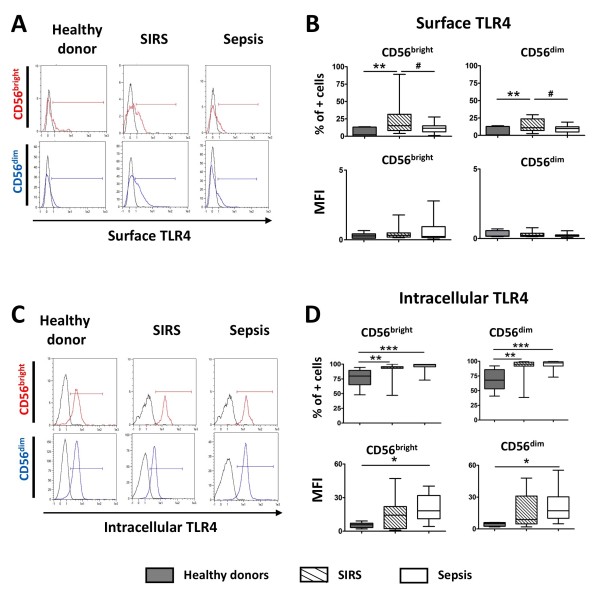
**Flow cytometry analysis of Toll-like receptor 4 (TLR4) expression in natural killer (NK) cell CD56^bright ^and CD56^dim ^subsets**. (**A**) Representative flow cytometry histogram of surface expression of TLR4 on CD56^bright ^and CD56^dim ^NK cell subsets for healthy volunteers, systemic inflammatory response syndrome (SIRS), and sepsis patients. Black line: isotype control; colored line: anti-TLR4. (**B**) Surface TLR4 expression shown as median and interquartile range in all three studied groups. (**C**) Representative flow cytometry histogram of intracellular expression of TLR4 for the same samples represented in (**A**). Black line: isotype control; colored line: anti-TLR4. (**D**) Intracellular TLR4 expression shown as median and interquartile range in all three groups. *P < 0.05; **P < 0.01; ***P < 0.001 for comparison with healthy donors. #*P *< 0.05 for SIRS versus sepsis patients. MFI, mean fluorescence intensity.

For TLR4, similarly to TLR2, surface expression was extremely low for healthy controls and patients in terms of MFI (Figure 3A-B). Nevertheless, in comparison to healthy controls and sepsis patients, SIRS patients showed an increase in the percentage of surface TLR4-positive cells for both NK subsets. In keeping with the trend observed for TLR2, a strong intracellular expression of TLR4 was noted for all three groups (Figure 3C-D). We found a significantly higher percentage of intracellular TLR4-positive NK cells in SIRS and sepsis patients for both CD56^bright ^and CD56^dim ^cells in comparison to healthy controls. In terms of MFI, an increase was again noted for both CD56^bright ^and CD56^dim ^NK cells of septic patients compared to healthy volunteers. In contrast to TLR2 and TLR4, no difference was found in the expression of TLR9, the receptor for CpG-DNA (Additional file 1), either in terms of percentage of positive cells or MFI.

### Sepsis increases the expression of CD69 on NK cells

**Figure 4:**
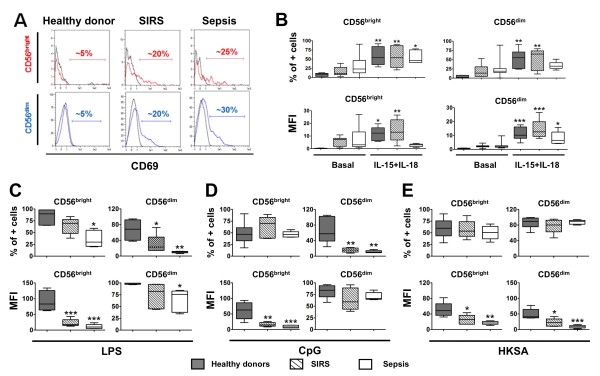
**Expression of CD69 on natural killer (NK) cell CD56^bright ^and CD56^dim ^subsets on freshly isolated cells or after *in vitro *cultures**. (**A**) Representative flow cytometry histograms of surface basal expression of CD69 on CD56^bright ^and CD56^dim ^NK cell subsets for healthy volunteers, systemic inflammatory response syndrome (SIRS), and sepsis patients. Black line: isotype control; colored line: anti-CD69. (**B**) CD69 expression in all three groups for freshly isolated NK cells (basal) or after overnight culture in the presence of IL-15 + IL-18; data are shown as median and interquartile range. CD69 expression expressed after overnight culture in the presence of IL-15 + IL-18 and lipopolysaccharide (LPS) (**C**), CpG (**D**) and heat-killed *S. aureus *(HKSA) (**E**); data are expressed as median and interquartile range. *P < 0.05; **P < 0.01; ***P < 0.001 for comparison between healthy donors and patients. MFI, mean fluorescence intensity.

We then measured the expression of CD69, an early marker of activation for various cell types, including NK cells [41,42]. As shown in Figure 4A-B, while CD69 was barely expressed on NK cells of healthy volunteers, it was increased and detected in sepsis and SIRS patients. Nevertheless, this increase in expression was not statistically significant for the percent positive cells or for MFI. In addition, we measured the expression of CX3CR1 and CD16. CX3CR1 is the receptor for the chemokine fractalkin, and is important for the recruitment of NK cells, especially the CD56^dim ^subset. This receptor has been shown to be involved in host defense during bacterial sepsis [43]. In addition, a study on human sepsis reported that CX3CR1 protein expression was decreased on patients' monocytes [44]. CD16 is the Fcγ receptor III, and is known to be modulated by viral infections [45]. Flow cytometry analysis comparing healthy controls, SIRS, and sepsis patients showed no differences in the expression of CX3CR1 or CD16 for either CD56^bright ^or CD56^dim ^NK cells subsets (data not shown).

### Impaired *ex vivo *upregulation of CD69 for NK cells from SIRS and sepsis patients

As mentioned previously, CD69 is not expressed on NK cells from healthy controls and is only expressed on 25 to 30% of NK cells from SIRS and sepsis patients. CD69 is upregulated upon stimulation, and we wondered if PAMPs or heat-killed *Staphylococcus aureus *(HKSA) would be able to enhance CD69 expression *ex vivo*. As shown in Figure 4B, IL-15 in combination with IL-18 induced upregulation of CD69 in all groups. While there was a more pronounced increase in the percentage of CD69 positive cells for CD56^bright ^subsets, the increase in MFI was higher in the CD56^dim ^subsets. The combination of these cytokines with LPS, CpG-DNA, or HKSA (Figure 4C-E, respectively) induced an even stronger expression of CD69 on both subsets for healthy controls (both percentage of positive cells and MFI). This was particularly noteworthy for CD56^dim^. Some increase was observed for SIRS NK cells in response to LPS, but in almost all cases (except MFI for CD56^dim ^for IL-15/IL-18 + CpG-DNA) the expression of CD69 on NK cells from patients remained significantly lower than levels observed for healthy controls. A similar conclusion can be drawn for sepsis patients. Only HKSA in the presence of IL-15 and IL-18 was able to increase the percentage of CD69 positive cells for CD56^dim ^subset. However, in terms of MFI, the expression of CD69 was again reduced on both NK subsets from patients.

### Impaired *ex vivo *IFN-γ production by NK cells in SIRS and sepsis patients

**Figure 5:**
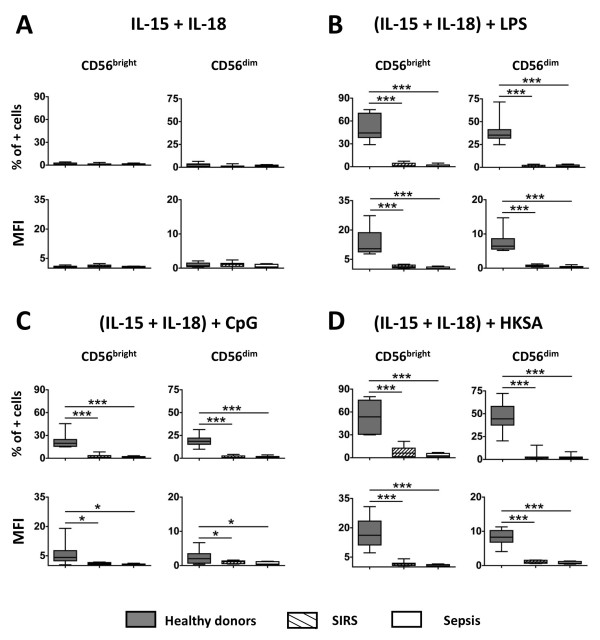
**Flow cytometry analysis of IFN-γ secretion in whole blood by CD56^bright ^and CD56^dim ^natural killer (NK) cell subsets after overnight *ex vivo *stimulation**. (**A**) IL-15 + IL-18; (**B**) IL-15 + IL-18 + LPS; (**C**) IL-15 + IL-18 + CpG-DNA; (**D**) IL-15 + IL-18 + heat-killed *S. aureus *(HKSA). Median and interquartile range are shown for each group. *P < 0.05; ***P < 0.001 for comparison between healthy donors and patients. MFI, mean fluorescence intensity.

The reactivity of NK cells to TLR agonists in terms of cytokine production has never been studied, especially in terms of IFN-γ production. We therefore studied the capacity of NK cells to produce IFN-γ *ex vivo *after stimulation with PAMPs or HKSA, and the combination of IL-15 and IL-18, two key cytokines for NK cell maturation and function. IFN-γ production was not observed for unstimulated blood alone (data not shown), nor when IL-15 and IL-18 were added in the absence of microbial activators (Figure 5A). Similarly, when NK cells were stimulated with PAMPs or HKSA alone, they were unable to produce IFN-γ (data not shown). Instead, the presence of IL-15/IL-18 and LPS, CpG-DNA or HKSA induced high levels of IFN-γ secretion by both subsets of NK cells for healthy controls (Figure 5B-D). In contrast, when the percentage of positive cells and MFI were measured in sepsis and SIRS patients, IFN-γ production was severely and significantly impaired (see Additional file 2 for representative flow cytometry histograms).

## Discussion

The present study aimed to further characterize the immune status of NK cells in patients with non-infectious SIRS or sepsis. In agreement with previous reports, we observed that the number of circulating NK cells was significantly decreased in SIRS and sepsis patients [30,31]. This decrease is the reflection of a general lymphopenia and may be due to the trafficking of NK cells to the sites of infection [46], or to apoptosis [47]. Interestingly, some reports suggest that NK cell percentages or counts are associated with outcome [48]. Our work further establishes that the decrease is similarly observed for both CD56^bright ^and CD56^dim ^NK cells subsets.

We characterized NK cells for the surface biomarkers CX3CR1 (fractalkine receptor), CD16 (FcγRIII), and CD69. While there was no significant modification of CX3CR1 and CD16 expression, we found a trend for an enhanced expression of the early activation marker CD69 *in vivo *after sepsis. However, as TLRs and CD69 stainings were not performed in the same tubes, we cannot compare TLR2 and TLR4 expression on CD69^- ^and CD69^+ ^NK cells. CD69 is upregulated upon activation by cytokines, TLR agonists or tumor cells. Indeed, sepsis is associated with an increase in many circulating cytokines and PAMPs (especially LPS). Previous reports showed that the upregulation of CD69 is a sensitive marker in neonatal sepsis [49] and reported the presence of circulating CD56^+^CD69^+ ^NK cells in sepsis [50].

For the first time, we explored TLR2, TLR4 and TLR9 expression on and in human blood NK cells during sepsis. First, our study revealed that NK cells from healthy human donors mainly express TLR2 and TLR4 intracellularly (the same expression pattern observed for TLR9). The intracellular expression of TLR2 and TLR4 has already been reported for other cells such as epithelial [51], endothelial [52], or even dendritic cells [53]. These intracellular TLRs are active, as reported by Hornef *et al. *[54] who showed the possible activation of epithelial cells after internalization of a TLR ligand. For NK cells, only a low percentage of both subsets express surface TLR2 and TLR4 at low density. A large part of the knowledge on TLR expression in NK cells from both human or mouse subjects has been acquired in studies based on mRNA detection, and remains controversial [22-24,39,40] However, mRNA detection is not equivalent to protein expression, which depends on translational and post-translational events that will confer the functionality and the right cellular localization. For this reason, we chose to address TLR2 and TLR4 expression in NK cells in terms of protein expression. We obtained very similar results with mouse spleen NK cells [55], suggesting that the intracellular expression of TLR2 and TLR4 is a general feature of naive NK cells present in a healthy environment. Our present study shows that this expression can be modified in human SIRS and sepsis. Most interestingly, the percentage of NK cells expressing intracellular TLR2 was significantly increased in sepsis for the CD56^dim ^subset. Similarly, the MFI of intracellular TLR2 in CD56^bright ^and CD56^dim ^subsets was significantly increased in sepsis patients compared to healthy controls. For TLR4, an increase in the percentage of NK cells positive for surface expression was only observed in SIRS patients. In contrast, intracellular TLR4 expression was significantly enhanced in sepsis patients and SIRS patients compared to healthy controls in both NK subsets. This is the first report showing a modulation of TLR expression in NK cells from ICU patients. To date, TLR2 and TLR4 expression has been mainly studied in monocytes, and most studies show enhanced surface expression of TLR2 in sepsis, while the data concerning TLR2 surface expression in trauma and SIRS patients or TLR4 expression are more controversial. It is still unclear which circulating mediators are able to modulate the TLR expression in NK cells. Nevertheless, it is of interest to note that the percentage of positive cells for surface TLR4 expression on NK cells allows discrimination of patients with sepsis and SIRS.

As a measure of the functionality of NK cells circulating in the blood of ICU patients, we show that NK cells undergo a dramatic alteration of their capacity to produce IFN-γ when exposed *in vitro *to TLR4 or TLR9 agonists in the presence of the accessory cytokines IL-15 and IL-18. This reduced production of IFN-γ occurs despite enhanced TLR4 expression in SIRS patients and unchanged TLR9 intracellular expression in all groups. The TLR2 agonist Pam3CysSK4 was tested on NK cells from healthy controls and on those from a few patients. Because in both cases the responsiveness was very low, we no longer used this TLR2 agonist in this study. In a similar fashion observed for NK cell response to purified PAMPs, the response to whole bacteria (HKSA) was profoundly altered. Although, altered IFN-γ production in response to LPS has already been reported in whole blood samples from sepsis patients [56], this is the first direct demonstration that IFN-γ production by NK cell subsets is altered in human sepsis and SIRS. Nevertheless, we addressed circulating NK cells, and their altered reactivity is not necessarily the reflection of that of their counterparts present in the tissues. Access to cells from compartments other than blood is much easier in animal models. In mice a decreased capacity of liver NK cells to produce IFN-γ in response to LPS has also been reported [57], and in this murine model of sepsis, Scott *et al*. hypothesized that IL-10 was responsible for the suppressed production of IFN-γ [58]. However, in a mouse model of sepsis, analyzing spleen NK cells, we recently showed that TGF-β, more than IL-10, was the deactivating cytokine [55]. To our knowledge only one group has performed studies with the human spleen. Their study reports an altered response to LPS, but does not address NK cell immune status [59]. Our results fit with the alteration of the cytotoxic activity already reported for NK cells from ICU patients. Indeed, reduced cytotoxic activity has been regularly reported for the past 25 years in patients with thermal injury [34,35] and more recently observed in both adult and newborn patients with sepsis [32,60]. Some reports also demonstrate that in many patients NK cells are unresponsive to IL-2 and IFN-α as assessed in terms of cytotoxicity.

It is important to note that NK cells from healthy donors need both signals, IL-15 and IL-18, and the presence of PAMPs, to produce IFN-γ *ex vivo*. In our experimental model, it is not yet possible to decipher whether NK cells from patients fail to respond to the cytokine cocktail (that is, IL-15 plus IL-18) used to favor their response to PAMPs and whole bacteria, or fail to respond to the microbial products themselves, or fail to respond to both. However, cytokines alone were able to upregulate the expression of CD69 in NK cells from SIRS and sepsis patients. Of note, in the presence of PAMPs or HKSA, NK cells from healthy controls showed an additional increase in CD69 expression *ex vivo *in terms of MFI. In almost all cases the expression of CD69 on NK cells from patients remained significantly lower than levels observed for healthy controls, suggesting that their NK cells are somehow refractory to CD69 upregulation by bacterial products *ex vivo*. This may suggest that the deficit in NK cell responsiveness for patients mainly occurs during the reaction to microbial agonists. We cannot exclude a role of accessory cells in whole blood via IL-12 production in the hyporesponsiveness observed for NK cells. However, we recently showed in a mouse model that purified NK cells are responsive to TLR agonists in the presence of IL-15/IL-18 and that they become tolerant to this *ex vivo *challenge following polymicrobial sepsis [55], which is in favor of a deactivation of NK cells themselves.

In animal models, including the injection of LPS, thermal injury, surgery or sepsis, altered cytotoxic activity has also been reported [61-63]. In these models, spleen NK cells or NK cells from other compartments, such as the lungs, also displayed a reduced capacity to produce IFN-γ [64]. It is worth mentioning that none of these murine models have addressed circulating NK cells, which are those studied in humans. This difference may explain why sometimes some discrepancies are found in the roles of NK cells in animal models of sepsis. Depending on the experimental model, NK cells have been reported to be beneficial or deleterious in fighting polymicrobial sepsis in the cecal ligation and puncture murine model. Among the factors that can contribute to NK cell dysfunction, altered macrophage accessory activity [25] and an immunosuppressive action of IL-10 [64], TGF-β and regulatory T cells (Tregs) [55] have been suggested. In addition, it is also possible that apoptosis, which may occur in surgery patients, and seems to take place also during sepsis, [59] contributes to alterations in NK cell functions. Indeed, IL-15 treatment of infected mice was shown to prevent NK cell apoptosis, and in cases of polymicrobial sepsis and pneumonia, treatment was associated with improved survival [65]. This observation suggests that NK cells with an appropriate immune status would be beneficial to counter the severe infectious processes observed in sepsis. This is also congruent with reports suggesting an association between low NK cell counts and poor patient outcome [48].

## Conclusions

In this study, we further extended the concept of CARS to human NK cells for both CD56^bright ^and CD56^dim ^subsets. We demonstrated a defect of NK cells for both CD69 upregulation and IFN-γ production *ex vivo *in response to accessory cytokines and PAMPs, or accessory cytokines and whole bacteria in sepsis and SIRS patients. Finally, we determined that expression of TLR2 and TLR4 is mostly intracellular in circulating human NK cells and that these receptors are upregulated differentially in and on NK cells of SIRS and sepsis patients, allowing discrimination of these two groups of patients.

## Key messages

• TLR2 and TLR4 are mainly expressed intracellularly in NK cells.

• Intracellular expression of TLR2 and TLR4 in both CD56^Dim ^and CD56^Bright ^NK cell subsets is enhanced during sepsis.

• CD69 expression is enhanced on NK cells from SIRS and sepsis patients but may still be futher increased by the addition of IL-15 plus IL18.

• Production of IFN-γ by circulating NK cells is altered in SIRS and sepsis patients.

## Abbreviations

ANOVA: analysis of variance; CARS: compensatory anti-inflammatory response syndrome; FCS: fetal calf serum; HKSA: heat-killed *Staphylococcus aureus*; IFN-γ: interferon-γ; IL: interleukin; LPS: lipopolysaccharide; MFI: mean fluorescence intensity; NK: natural killer; NOD2: oligomerization domain receptor 2; PAMP: pathogen-associated molecular pattern; PBS: phosphate-buffered saline; SIRS: systemic inflammatory response syndrome; TLR: Toll-like receptor; Tregs: regulatory T cells.

## Competing interests

The authors declare that they have no competing interests.

## Authors' contributions

FSFG and MP performed the experiments, analyzed the data, and prepared the figures. FP and BM supervized the inclusion of the patients and included the clinical parameters. JMC initiated the project and wrote the manuscript. MAC supervised the study and wrote the manuscript. All authors have read and approved the manuscript for publication.

## Supplementary Material

Additional file 1 **Expression of Toll-like receptor 9 (TLR9) in CD56^bright ^and CD56^dim ^natural killer (NK) cells subsets**. A figure showing (**A**) Representative flow cytometry histogram of TLR9 intracellular expression in healthy donors, systemic inflammatory response syndrome (SIRS), and sepsis patients. Black line: isotype control; color line: anti-TLR9. (**B**) Median and interquartile range for each group. We found no significant difference between groups. MFI, mean fluorescence intensity. Click here for file

Additional file 2 **Representative histograms of IFN-γ secretion analyzed by flow cytometry for CD56^bright ^and CD56^dim ^natural killer (NK) cell subsets for healthy donors, systemic inflammatory response syndrome (SIRS), and sepsis patients**. A figure showing the result after overnight *ex vivo *stimulation in whole blood by lipopolysaccharide (LPS), CpG oligonucleotide or heat-killed *Staphylococcus aureus *in the presence of IL-15 + IL-18. Black line: unstimulated cells; colored line: IFN-γ positive cells. Click here for file
