# Supplementary figures and images for: Toll-like receptors expression and interferon-γ production by NK cells in human sepsis
Source: Crit Care. 2012 Oct 25;16(5):R206. doi: 10.1186/cc11838 (PMC3682310; doi:10.1186/cc11838)

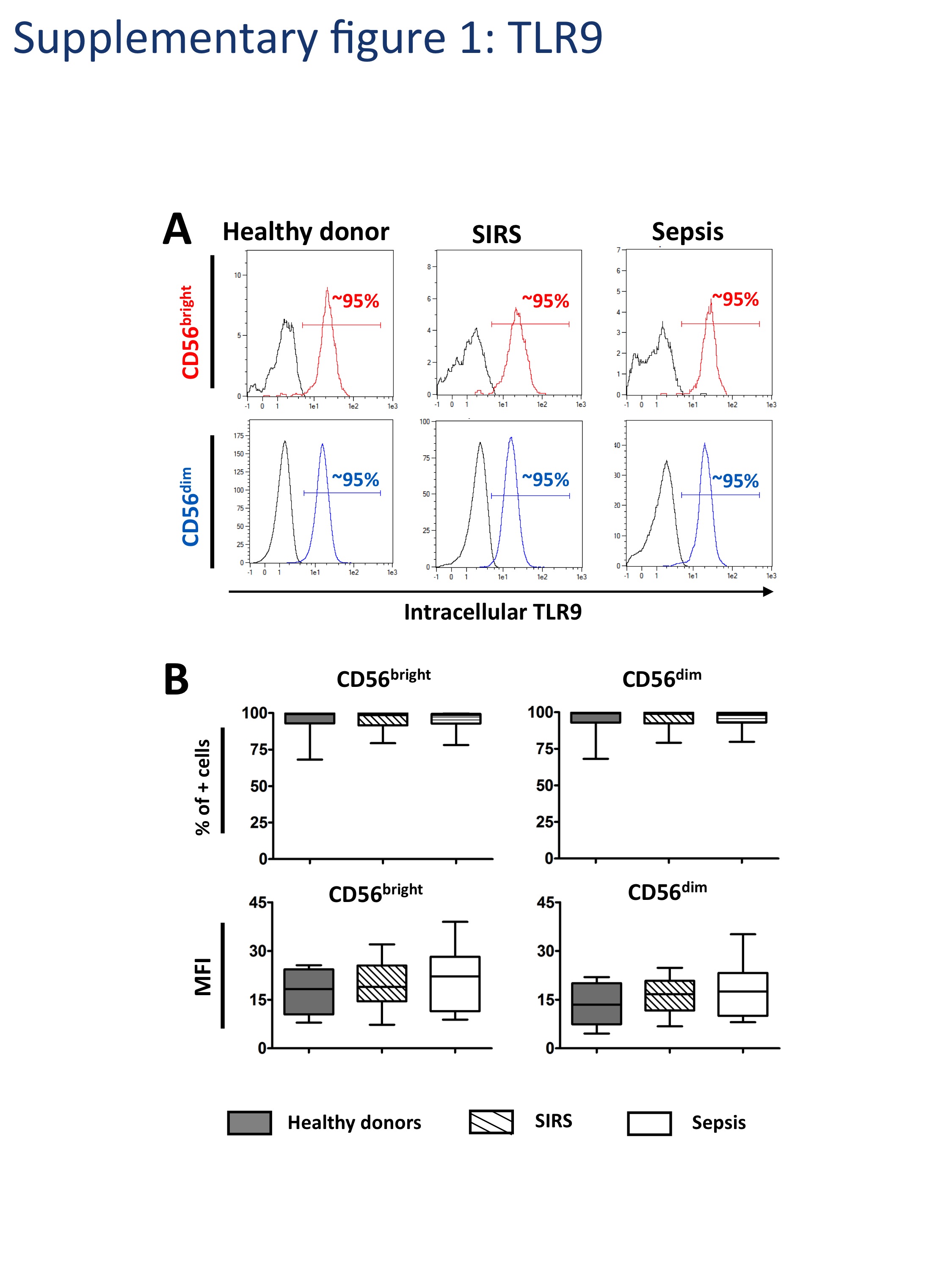

Supplement: Additional file 1 — Expression of Toll-like receptor 9 (TLR9) in CD56bright and CD56dim natural killer (NK) cells subsets. A figure showing (A) Representative flow cytometry histogram of TLR9 intracellular expression in healthy donors, systemic inflammatory response syndrome (SIRS), and sepsis patients. Black line: isotype control; color line: anti-TLR9. (B) Median and interquartile range for each group. We found no significant difference between groups. MFI, mean fluorescence intensity. [file cc11838-S1.JPEG]

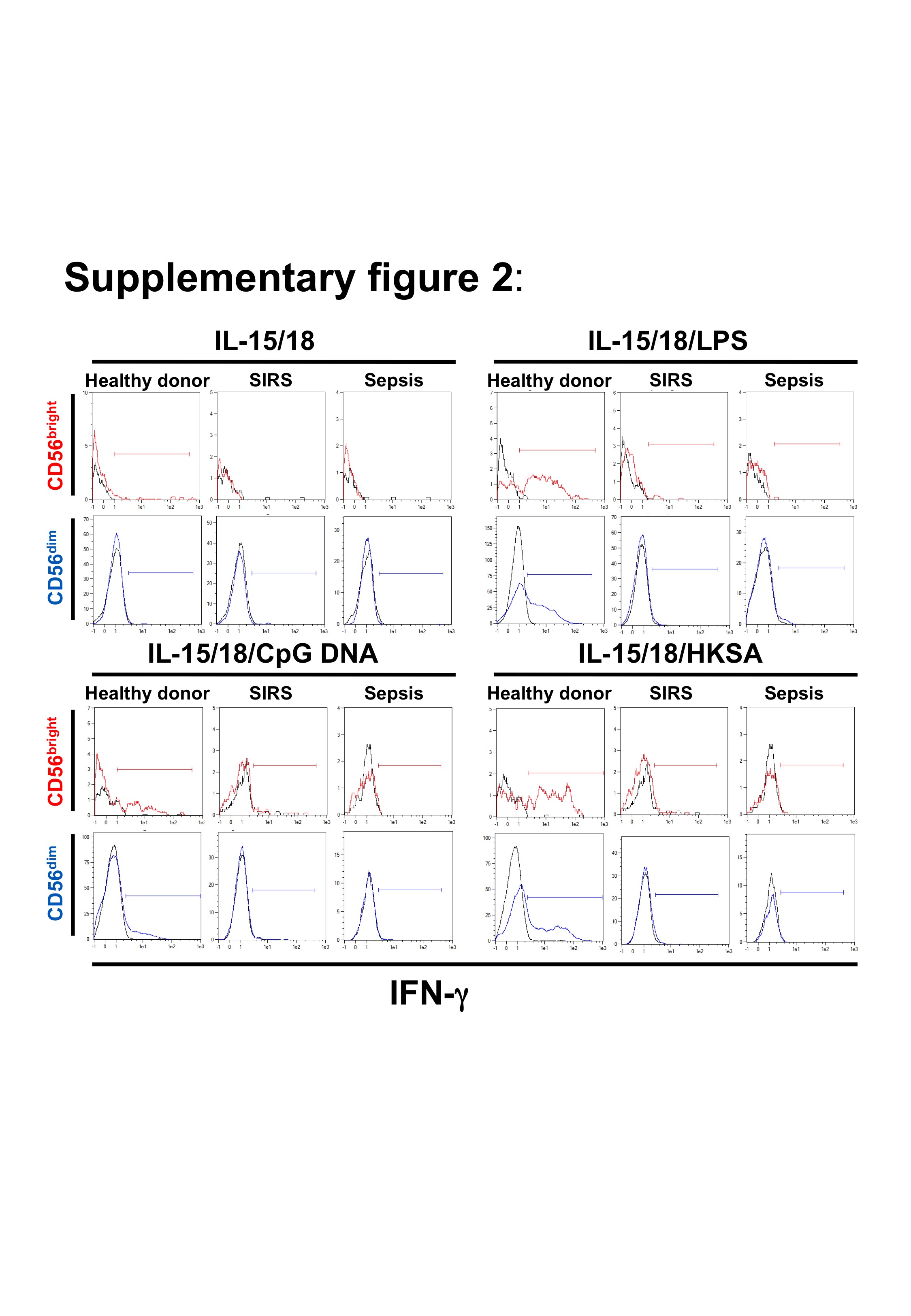

Supplement: Additional file 2 — Representative histograms of IFN-γ secretion analyzed by flow cytometry for CD56bright and CD56dim natural killer (NK) cell subsets for healthy donors, systemic inflammatory response syndrome (SIRS), and sepsis patients. A figure showing the result after overnight ex vivo stimulation in whole blood by lipopolysaccharide (LPS), CpG oligonucleotide or heat-killed Staphylococcus aureus in the presence of IL-15 + IL-18. Black line: unstimulated cells; colored line: IFN-γ positive cells. [file cc11838-S2.JPEG]
